# Can pharmaceutical pollution alter the spread of infectious disease? A case study using fluoxetine

**DOI:** 10.1098/rstb.2022.0010

**Published:** 2023-03-27

**Authors:** Lucinda C. Aulsebrook, Bob B. M. Wong, Matthew D. Hall

**Affiliations:** School of Biological Sciences, Monash University, Melbourne Victoria 3800, Australia

**Keywords:** host–parasite interactions, *R*_0_, epidemic, parasite reproduction, global change

## Abstract

Human activity is changing global environments at an unprecedented rate, imposing new ecological and evolutionary ramifications on wildlife dynamics, including host–parasite interactions. Here we investigate how an emerging concern of modern human activity, pharmaceutical pollution, influences the spread of disease in a population, using the water flea *Daphnia magna* and the bacterial pathogen *Pasteuria ramosa* as a model system. We found that exposure to different concentrations of fluoxetine—a widely prescribed psychoactive drug and widespread contaminant of aquatic ecosystems—affected the severity of disease experienced by an individual in a non-monotonic manner. The direction and magnitude of any effect, however, varied with both the infection outcome measured and the genotype of the pathogen. By contrast, the characteristics of unexposed animals, and thus the growth and density of susceptible hosts, were robust to fluoxetine. Using our data to parameterize an epidemiological model, we show that fluoxetine is unlikely to lead to a net increase or decrease in the likelihood of an infectious disease outbreak, as measured by a pathogen's transmission rate or basic reproductive number. Instead, any given pathogen genotype may experience a twofold change in likely fitness, but often in opposing directions. Our study demonstrates that changes in pharmaceutical pollution give rise to complex genotype-by-environment interactions in its influence of disease dynamics, with repercussions on pathogen genetic diversity and evolution.

This article is part of the theme issue ‘Infectious disease ecology and evolution in a changing world’.

## 1. Introduction

Human-induced rapid environmental changes pose a serious threat to global biodiversity. Not only are ecosystems being impacted by an array of anthropogenic challenges that can directly affect population persistence in a wide range of species [1], such challenges can also influence the capacity of organisms to cope with additional threats or stressors [2,3]. One key environmental stressor confronting animals is pathogens, which are ubiquitous across ecosystems, and dramatically alter host health and population dynamics owing to the fitness costs pathogens impose on their hosts upon infection [4]. Interactions between pathogens and their hosts have been found to be disrupted or modified by numerous anthropogenic stressors; for example, contact with pesticides can increase trematode infections in amphibians [5,6], while exposure to heavy metals is associated with increased mortality in a range of species, from snails to fish [7,8]. Clearly, widespread anthropogenic challenges, such as chemical pollution, can have a profound impact on host–pathogen dynamics.

One group of chemical pollutants of growing concern is pharmaceuticals, with more than 600 different products detected in waterways, lakes and rivers around the world [9,10]. Pharmaceuticals typically enter the environment by being consumed and then excreted still in a bioactive form [11]. Wastewater treatment is typically inadequate for removing these bioactive pollutants, so sewage effluent is a primary source of environmental contamination [11]. Evidence suggests that many pharmaceutical pollutants are slow to degrade [11] and can bioaccumulate [12], with potentially dire consequences for exposed organisms [13], from feminization of male fish [14–16] to death in vultures [17]. With the exception of antimicrobials such as antibiotics [18,19], however, and in contrast to what is known about toxicants such as pesticides and heavy metals (e.g. [4–7]), the impact of pharmaceutical pollutants on disease dynamics remains largely unknown (see [18]).

Our limited knowledge of how pharmaceuticals affect disease dynamics is concerning because pharmaceutical pollutants can impact organisms in unique ways compared with other environmental contaminants. Firstly, pharmaceutical pollutants frequently exert non-monotonic responses, where lower doses can have larger effects than higher doses (e.g. [20–22]). This may be because these drugs are designed to act effectively at selective low doses, and their intended receptors become desensitized at higher doses [23]. Secondly, since pharmaceuticals are typically designed to have therapeutic benefits, it is possible that exposure of non-target organisms to these pollutants could even have positive effects on fitness, such as growth and reproduction [20,24]. By contrast, traditional forms of chemical pollution such as pesticides and heavy metals typically reduce individual health and performance [25–27].

An understanding of the effects of pharmaceutical pollutants on host–parasite interactions begins by considering how varying concentrations of a pollutant may influence an outbreak of infectious disease. The likelihood that a pathogen will spread through a host population is commonly evaluated by estimating the basic reproductive number of a pathogen, known as *R*_0_ [28]. Ecological studies of infectious disease, in particular, have explored the sensitivity of *R*_0_ to different forms of environment change (e.g. resource availability: [29], temperature: [30,31]), with values above unity indicating that pathogen population will increase in size. Importantly, *R*_0_ depends on both the characteristics of infected carriers (e.g. spore loads) and the overall density of the host population [28], something that pharmaceutical pollutants may uniquely enhance. Like any form of pollution or stress, however, it is unlikely all host or pathogen genotypes within a population will be affected by pharmaceutical pollutants in the same way [32]. How the relative fitness of each genotype in a population changes in response to pharmaceutical pollution will also determine the evolutionary consequences of any pollution event. If a pollutant results in changes in relative differences among genotypes, then the rate of host or pathogen evolution may be slowed or enhanced (scale G × E [33]). Alternatively, if pollution results only in a change in the rank order of host or pathogen genotypes, then the maintenance of genetic diversity may result, as different genotypes will be favoured when exposed [34].

An emerging pharmaceutical pollutant that may shape the spread of infectious disease is fluoxetine (marketed as Prozac), one of the most prescribed antidepressants in the world [35], as well as a persistent (half-life of 68 days under pH 7 and light conditions [36]) and commonly detected pollutant in the environment ([12]; see also https://www.umweltbundesamt.de/en/database-pharmaceuticals-in-the-environment-0). As a serotonin reuptake inhibitor, fluoxetine works by targeting the serotonin transporter (5-HTT or SERT) [37], which is evolutionarily conserved in a wide range of species, including both vertebrate and invertebrate taxa [38]. Fluoxetine exposure has been found to have a variety of effects on numerous aquatic organisms, such as disrupting ecologically important behaviours and life-history traits in a variety of vertebrate [22,39] and invertebrate [20,21,40,41] species. By contrast, the effects of fluoxetine on disease and host–pathogen interactions have been largely overlooked. Among the notable exceptions, Pax *et al*. [42] found that fluoxetine decreased the muscular activity of the water-borne human parasite *Schistosoma mansoni*, and Dechaumes *et al.* [43] found that injecting fluoxetine into SARS-CoV2-infected cells inhibited virus activity. Both studies, however, were designed to investigate the therapeutic role of fluoxetine to inhibit human disease and, as a result, used fluoxetine doses that are several orders of magnitude higher than those detected in the wild. Surprisingly, no study has investigated whether fluoxetine pollution, or indeed other psychoactive pharmaceutical pollutants, might affect the spread of infectious disease at ecologically relevant concentrations.

Our study assesses the impacts of fluoxetine on the dynamics of infectious disease using the freshwater filter-feeding crustacean *Daphnia magna* and its bacterial pathogen *Pasteuria ramosa* as our study system*.* The host–parasite dynamics of this system are well characterized, with infections typically resulting in a severe loss of fecundity and lifespan [44–46]. *Daphnia* and *P. ramosa* are frequently used as a model for studying ecology and evolution of disease, as both host and pathogen fitness has been shown to be influenced by both host and pathogen genotype, as well as environmental factors (reviewed in [45]). *Daphnia* is also a model system in ecotoxicology (e.g. [47]), as they are found in a range of aquatic systems, including those that are strongly subject to wastewater pollutants. More specific to our study, environmental levels of fluoxetine have been found to affect the fecundity and growth of *Daphnia* [20], both of which are expected to have repercussions for disease spread.

Here, we combine experiments with epidemiological models to assess the impact of four concentrations of fluoxetine and six pathogen genotypes on the likelihood an outbreak of infectious disease might occur. The fluoxetine concentrations chosen capture the lower and upper limits of fluoxetine detected in the environment [48] as well as levels used in acute toxicology studies (e.g. [49]). First, we examine whether fluoxetine alters the fecundity, lifespan and intrinsic growth rates of uninfected hosts in order to assess how this pollutant may alter the supply of susceptible hosts in a population. Second, we determine whether fluoxetine exposure alters the characteristics of infected individuals by measuring traits such as infection rate, spore loads, and mortality. Finally, using the traits of both susceptible hosts and infected individuals, we parameterize a model to ascertain whether fluoxetine will alter the rate at which new infections are created (i.e. transmission), and the potential for a pathogen to spread through a susceptible population (*R*_0_) under each condition. With this design, we aim to examine whether the non-monotonic effects of pharmaceutical pollutants are likely to extend to the outcome of infectious disease, as well as explore their influence on the potential ecology and evolution of host–parasite dynamics in natural populations.

## 2. Methods

### (a) Study system and between-host model overview

*Daphnia magna*, while capable of reproducing sexually, most frequently reproduces asexually via cyclic parthenogenesis, with the density of a population predicted to depend on the intrinsic growth rates of the hosts and the carrying capacity of the population [29,50]. *Daphnia* can become infected with *P. ramosa* through ingesting free-living spores present in the water column or sediment [45]. The infection becomes chronic if it is not cleared shortly after infection, and results in a severe loss of lifespan and fecundity (i.e. virulence), and an increase in body size [44–46,51]. *Pasteuria ramosa* spores proliferate within the host until host death, whereupon the spores are released from the cadaver. These spores may then be ingested by other *Daphnia*, or contribute to the pool of free-living spores in the environment [45].

We can capture the features of this system using a simple model of between-host dynamics (see [52]; adapted from [53]; [29,50]). Using *S*, *I*, *C* and *F* to denote the densities of susceptible hosts, infected hosts, cadavers of infected hosts and free-living spores of the pathogen, the epidemiological model we use is

2.1 $$\frac{dS}{dt}=b(S+mI)(1-k(S+I))-\mu S-\beta SF,$$

2.2 $$\frac{dI}{dt}=\beta SF-DI,$$

2.3 $$\frac{dC}{dt}=DI-\delta^{C}C$$

2.4 $$\mathrm{and}\;\frac{dF}{dt}=\rho \omega C-\delta^{F}F,$$

where susceptible hosts increase in a density-dependent manner (controlled by maximum birth rate, *b*, and the strength of density-dependence, *k*) and infection leads to a reduction in fecundity (0 ≤ *m* ≤ 1) owing to castration. All individuals suffer a constant *per capita* mortality rate (*μ*), with an additional rate of pathogen-induced mortality experienced by infected individuals (i.e. virulence, *v*). Term *D* thus captures the rate at which infections end owing to host death (i.e. *D* = *μ* + *v*). Susceptible hosts become infected at a rate influenced by the ingestion of free-living spores from the environment pool and the per-spore capacity of a pathogen to establish successfully in a host (environmental transmission, *β*). All cadavers release spores into the environment at a rate that is proportional (with coefficient *ρ*) to the spore load at the time of host death (*ω*), and decay at rate *δ^C^*. Finally, *δ^F^* is the loss rate of spores from the environmental pool through death or degradation.

### (b) Experimental methods: pathogen and fluoxetine exposure

From day 1, *D. magna were* exposed to one of four nominal fluoxetine concentrations: 30 ng l^−1^ (low)—representing the concentrations detected at surface waters, 300 ng l^−1^ (medium)—representing concentrations at wastewater outlets [48], 3000 ng l^−1^ (high)—representing concentrations typically used in acute toxicology studies [49], or 0 ng l^−1^—freshwater control. For each concentration, animals from a single *Daphnia* genotype BE-OHZ-M10 (derived from a single clone from Belgium) were exposed to 25 000 spores from one of six *P. ramosa* genotypes (C1, C14, C18, C19, C20 and C24) on day 4, or kept as an unexposed control. Details of the standard culturing conditions and infection process are outlined in the electronic supplementary material. Our experiment thus used a full factorial design with 25–30 individuals per treatment (6 pathogens + uninfected controls × 4 fluoxetine treatments). The slight variation in number of individuals per treatment was due to differences in infection rate, as well as survival and handling errors.

During the experiment, *Daphnia* were checked daily for survival, and any dead individuals were frozen in 0.5 ml reverse-osmosis water at −20°C for later assessment of spores. Offspring production was counted biweekly at each water change, allowing the estimation of fecundity at 12 weeks and lifetime fecundity for each animal. Fortnightly water samples were taken for the analysis of fluoxetine concentrations, with measured concentrations of 12.3 ± 1.3 ng l^−1^ for low, 114.0 ± 9.9 ng l^−1^ for medium and 959 ± 75 ng l^−1^ for high (see electronic supplementary material). Following the deaths of all experimental animals, the number of mature spores was counted by crushing each *Daphnia* cadaver, except those that had been determined to be uninfected based on visual inspection (following [45]), and using a Neubauer improved counting chamber to count spores for two independent samples of each suspension. If no mature spores were present, *Daphnia* were categorized as uninfected.

### (c) Quantifying the characteristics of individual unexposed and infected animals

All analyses were conducted in R (v. 4.2; R Development Core Team, www.R-project.com). We began by investigating the effect of fluoxetine treatment on fecundity at 12 weeks, lifetime fecundity, lifespan and the intrinsic growth of susceptible hosts. Intrinsic rates of increase per individual (*r*) were calculated by solving the Euler–Lotka equation, using the timing and number of offspring, as well as timing of deaths (following [20,54]). Each trait was analysed by fitting a linear model with fluoxetine treatment as a fixed effect (four levels: control, low, medium and high) with significance (*α* = 0.05) assessed via an analysis of variance test.

We next explored the influence of fluoxetine on the characteristics of infected individuals, namely infection rates, spore loads at host death, and lifespan. For each trait, we analysed the fixed effects of fluoxetine treatment (as above), pathogen genotype (six levels: C1 to C24), and their interaction via linear or generalized linear (for infection rates only, with a binomial link function) models, with significance of each term again assessed via an two-factor analysis of variance test (type III, car package: [55]). The *emmeans* package [56] was then used to predict the infection probability and standard errors for each pathogen and fluoxetine combination. The same package was also used to generate any *post hoc* pairwise tests.

### (d) Deriving metrics of pathogen fitness and the likelihood of an outbreak of infectious disease

To investigated how fluoxetine exposure might impact the spread of infectious disease through a host population, we obtained two metrics of pathogen fitness from our model—one that is solely dependent on the life-history characteristics of a pathogen, and another which is also linked to the dynamics of the susceptible host population. First, we further simplified the above model (equations (2.1)–(2.4)) by assuming that the dynamics of decaying cadavers and spores in the environment are fast relative to the other epidemiological dynamics. When this is true, then d*C*/d*t* = 0 and d*F*/d*t* = 0, giving $C=(\mu +v)I/\delta^{C}$ and $F=p\omega C/\delta^{F}$, and the model can be rewritten as

2.5 $$\frac{dS}{dt}=b(S+cI)(1-k(S+I))-\mu S-\beta \phi \omega DSI$$

and

2.6 $$\frac{dI}{dt}=\beta \phi \omega DSI-DI,$$

where $\phi =p/\delta^{C}\delta^{F}$ and describes the rate at which spores are released into the environment, relative to the rate at which cadavers and spores are removed. From this simplified model (equations (2.5) and (2.6)), we can derive a composite transmission rate, *σ* = *βϕωD*, which describes the *per capita* rate at which new infections are created based only on the characteristics of infected individuals. This term will increase with an increase in environment transmission rates (*β*), spore loads at host death (*ω*) or the rate at which infections end owing to host mortality (*D*). It also depends on the effective production rate of free-living environmental spores (*ϕ*).

By returning to the expanded model (equations (2.1)–(2.4)), we can also estimate the basic reproductive number, *R*_0_ [28], for each treatment combination. We obtained the expression for *R*_0_ using the next-generation matrix approach [57]. From our model, *R*_0_ is

2.7 $$R_{0}=\left(\frac{b-\mu }{bk}\right)\phi \omega \beta ,$$

where $\phi =p/\delta^{C}\delta^{F}$ as above. This metric is dependent on the growth of susceptible hosts in the absence of disease (i.e. (*b* − *μ*)/*bk*), and three epidemiological traits, as described by *ϕωβ*. *R*_0_ will increase if there are increases in host birth rate (*b*), the environmental transmission rate (*β*), or spore loads at host death (*ω*). *R*_0_ will decrease when there are increases in baseline host death rates (*μ*), or the strength of density-dependence on host birth rate (*k*), or a reduction in the effective rate of production of free-living environmental spores (*ϕ*).

### (e) Parameterization of transmission and *R*_0_

To calculate transmission (*σ*) and *R*_0_ for each fluoxetine and pathogen combination we calculated the relevant components of the models using data from the animals that we observed from birth until death. For transmission, direct estimates of spore loads at host death (*ω*) were available for each infected individual and we estimated the rate at which infections end owing to host death (*D*) as the inverse of host lifespan (1/days). The environmental transmission rate (*β*) was estimated using the numbers of infected and uninfected individuals from each treatment, where the probability of remaining uninfected (*P*) depends on the density of pathogen spores (*Z*, 25 000 per 20 ml) and the length of the infection period (*t*, 4 days), such that *P* = *e*^−*β*^*^Zt^* (following [54,58]). For *R*_0_, we estimated the *per capita* mortality rate (*μ*) as the inverse of host lifespan (1/days) for the unexposed (susceptible) hosts, and then host birth rate (*b*) as the sum of the intrinsic rate of increase (*r*, estimated directly, see above) and *μ* for the unexposed hosts (*b* = *r* + *μ*, [50,54,58]).

For all derived traits we used the Stan modelling language [59] as implanted via the *CmdStanR* package v. 0.5.2 [60]) in R, to calculate Bayesian posterior distribution estimates for each metric in turn (see also [54,58]). We used the default sampling settings of Stan and semi-informative priors that follow the appropriate distributions for each trait. In all following analyses, we arbitrarily set *ϕ* = 0.01 (following [52]) and *k* = 0.01 (following [50,54]) as they were not measured in this experiment. To calculate our indicator of the potential for disease spread (*σ* and *R*_0_) we incorporated the posterior samples of each of the metrics described above into our derived equations, and then characterized the resulting distribution via its mean and 90% uncertainty intervals (capturing that 95% of the mass in this interval is above or below the outer values).

## 3. Results and discussion

### (a) Fluoxetine has limited impact on the supply of susceptible hosts

**Figure 1.**
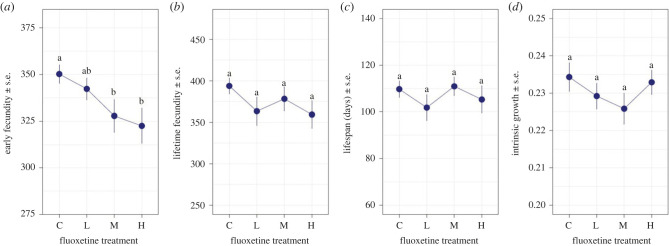
The effect of fluoxetine exposure on the supply of susceptible hosts, as measured by (*a*) early fecundity (as measured for the first 12 weeks), (*b*) lifetime fecundity, (*c*) lifespan, and (*d*) intrinsic growth rates (*r*) of *Daphnia magna* that have not been exposed to *Pasteuria ramosa*. Freshwater control (0 ng l^−1^), low (measured concentration: 12 ng l^−1^), medium (114 ng l^−1^) and high fluoxetine (959 ng l^−1^) treatments are denoted as C, L, M and H, respectively*.* Points represent treatment means (± s.e.). Lowercase letters indicate significant groupings by *post hoc* comparisons, where shared letters indicate that groups are not significantly different from each other (*p* < 0.05). (Online version in colour.)

The spread of disease depends on both the dynamics of infected hosts and the supply of susceptible hosts in a population [28]. We therefore first examined how fluoxetine pollution would influence the supply of susceptible hosts, by examining the fitness consequences of fluoxetine for animals that were not exposed to a pathogen. We found that increasing the concentration of fluoxetine led to a significant decline in the fecundity of the unexposed animals in the first 12 weeks (figure 1*a*, *F*_3,81_ = 2.932, *p* = 0.038). These effects, however, diminished over the lifetime of the *Daphnia*, as there were no overall differences in lifetime fecundity (figure 1*b*, *F*_3,91_ = 1.139, *p* = 0.337) and lifespan (figure 1*c*, *F*_3,91_ = 0.762, *p* = 0.518) across fluoxetine treatments. As a result, there was also no significant difference in the intrinsic growth rates of unexposed *Daphnia* raised in each of the different fluoxetine concentrations (figure 1*d*, *F*_3,91_ = 1.078, *p* = 0.363).

Our results suggest that fluoxetine has the capacity to affect reproduction of *Daphnia*, but that these effects are most pronounced early in the exposure period rather than over the lifetime of the *Daphnia*. Fluoxetine-induced fecundity effects have previously been observed in *Daphnia* [20,41] as well as other invertebrates [40,61], but these studies typically measure the responses in the weeks following initial exposure, rather measuring the lifetime reproductive success. It is also likely that the effects of fluoxetine will be felt in other aspects of a host's life-history, such as time to maturity, growth, or adult body size, as we have previously shown that the strength and form of any response to fluoxetine is highly trait-specific [20]. Nonetheless, based on these results, fluoxetine exposure appears unlikely to significantly alter disease dynamics purely through changes in the supply of susceptible hosts.

### (b) Evidence for the non-monotonic influence of fluoxetine on infected individuals

**Figure 2.**
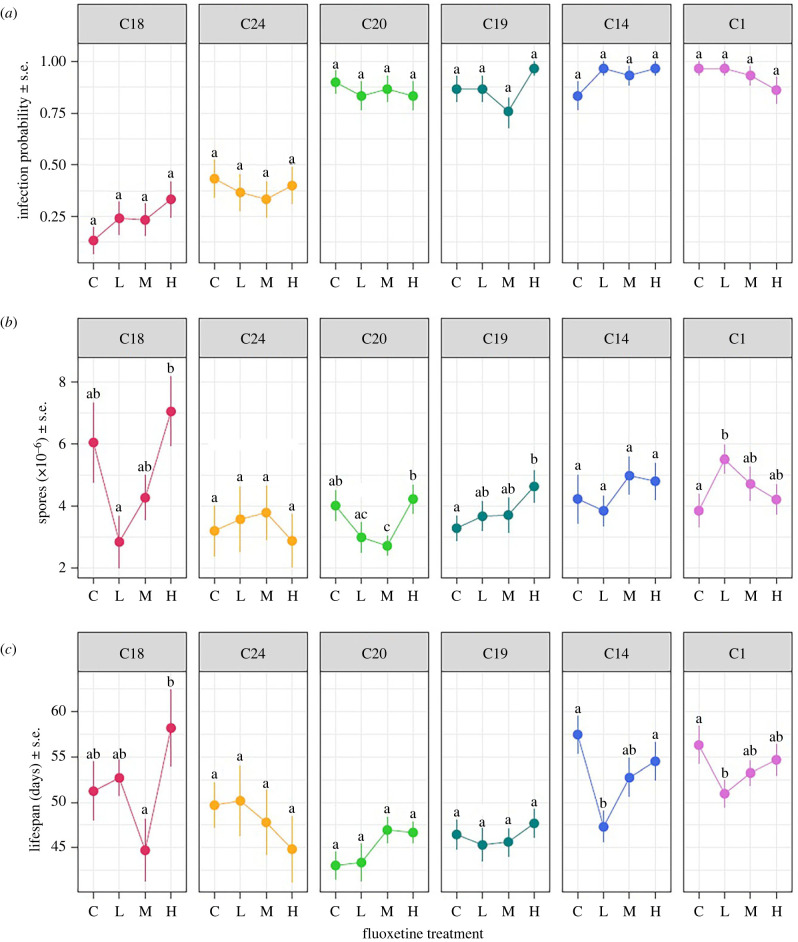
The effect of fluoxetine exposure on (*a*) the probability of successful infection, (*b*) mature spore loads per infected individual, and (*c*) lifespan of infected hosts for six different pathogen genotypes (C18, C24, C20, C10, C14 and C1). Freshwater control (0 ng l^−1^), low (measured concentration: 12 ng l^−1^), medium (114 ng l^−1^) and high fluoxetine (959 ng^−1^) treatments are denoted as C, L, M and H, respectively. Points represent treatment means (± s.e.). Lowercase letters indicate significant groupings by *post hoc* comparisons conducted separately for each pathogen genotype, where shared letters indicate that groups are not significantly different from each other (*p* < 0.05). (Online version in colour.)

We next compared the influence of fluoxetine pollution on the properties of infected hosts. We found that the probability of a pathogen successfully infecting a host depended on pathogen genotype alone, and was not significantly influenced by fluoxetine exposure (table 1 and figure 2*a*). In this case, C24 and C18 had far lower infection probabilities than other pathogen genotypes, which is relatively consistent with previous studies at comparable doses (e.g. [62]). Our results, therefore, do not indicate that fluoxetine exposure alters a host's susceptibility to infection. In general, chemical pollutants are believed to increase a host's susceptibility of disease [4,63] via two mechanisms: one where exposed animals are more vulnerable to infection owing to the energetic cost of detoxification [63]; the other where the chemical acts in a manner that directly interferes with their immune response (e.g. [5,6]). Based on the minimal effects seen here on unexposed hosts (above and figure 1), and the lack of change in host susceptibility, it appears that environmental levels of fluoxetine are unlikely to be associated with costly detoxification or impaired immune function with regards to pathogen clearance (cf. [64]).

**Table 1.** Effects of pathogen genotype, fluoxetine treatment, and their interaction on the probability of successful infection, mature spore loads, and the lifespan of infected *Daphnia magna*. Analysis was performed using a general linear model for infection probability, and linear models for spore loads and lifespan. *p < 0.05, **p < 0.01, ***p < 0.001.

trait	term	*F* or *χ*^2^	d.f.	*p*
infection probability	pathogen	280.292	5	<0.001***
	fluoxetine	2.054	3	0.561
	pathogen × fluoxetine	16.751	15	0.334
spore loads	pathogen	3.635	5, 478	0.003**
	fluoxetine	1.944	3, 478	0.122
	pathogen × fluoxetine	1.747	15, 478	0.039*
lifespan	pathogen	15.670	5, 478	<0.001***
	fluoxetine	2.313	3, 478	0.075
	pathogen × fluoxetine	1.762	15, 478	0.037*

By contrast, once a host was infected, both the degree of pathogen proliferation (as estimated by spore loads) and the duration of the infection (as estimated by host lifespan) depended on an interaction between the concentration of fluoxetine and the genotype of the invading pathogen (table 1). Both of these traits have important implications on the spread of disease, as the generation of new infections is contingent on pathogen proliferation (i.e. the mass-action principle, [65]), while increased mortality can allow faster host turnover [52]. In both fitness components, variation among pathogen genotypes, as well as the interaction between genotype and environment, is a common feature of this disease system [44,52] and many others (reviewed in [32]). Here we show fluoxetine, and thus potentially other pharmaceutical pollutants, gives rise to similar complex interactions.

This interaction was driven by differences in the outcome of infection between pathogen genotypes, where variation in both the intensity and form of responses occurred. For some genotypes, such as C19 and C24, the spore load and lifespans of infected hosts were mostly unaffected by the change in fluoxetine concentration (figure 2*b*, *c*). For most genotypes, however, we observed non-monotonic responses in at least one outcome of infection, whereby the greatest change in spore loads or host lifespan occurred at intermediate fluoxetine concentrations rather than the highest concentration. The form of the responses varied between genotypes (figure 2*b*, *c*). For pathogen C18, for example, both spore load and lifespan were minimized at intermediate concentrations, whereas for pathogen C1 there were opposing non-monotonic effects on spore load and lifespan, where spore loads increased at intermediate concentrations while lifespan decreased at these concentrations. For other pathogen genotypes, fluoxetine resulted in changes of only one outcome of infection (spore loads in C20, host lifespan in C14).

Our results demonstrate that the non-monotonic responses induced by fluoxetine are not restricted to previously reported behavioural and life-history traits (e.g. [20–22]), but extend to traits related to the interaction between a focal organism and its pathogens. This contrasts with many other documented interactions between pollutants, hosts and pathogens, where generally an increased concentration of the pollutant is associated with more severe effects (e.g. [5,7,64,66]). This distinction is important, as non-monotonic responses in infected individuals mean that ecosystems exposed to low levels of fluoxetine pollution will not necessarily be less affected than those exposed to higher concentrations. If true for other pharmaceutical pollutants, this means that management strategies for mitigating the effects of these pollutants (e.g. advanced oxidation processes, reviewed in [67]; activated carbon, reviewed in [68]) should not only be targeted towards regions with the most extreme cases of pharmaceutical pollution.

In addition, our results highlight the importance of considering secondary stresses such as pathogens when attempting to discern the full effects of pharmaceutical pollutants on population health and function. This is best highlighted by comparing the influence of fluoxetine on host lifespan in the presence and absence of a pathogen (figure 1*c* versus figure 2*c*). Only when a host was infected did we see the non-monotonic effects of fluoxetine expressed. This demonstrates that even when the effects of fluoxetine on individuals appear subtle or undetectable, other stressors in the environment may activate their effects. For many populations, the full effect of fluoxetine and pharmaceutical pollutants may thus only manifest when the effects of secondary stressors are realized. Although not all other stressors necessarily exacerbate the effect of fluoxetine (e.g. temperature [20]), such synergistic interactions between pollutants and other environmental stressors are being increasingly reported (reviewed in [2,69]), emphasizing that assessing the effects of any pollutant in isolation may overlook its true impact.

### (c) The ecological and evolutionary consequences of fluoxetine for pathogen invasion

Our results indicate that the properties of unexposed hosts that underlie the supply of susceptible hosts are largely robust to fluoxetine exposure, while the influence of fluoxetine on infected individuals is specific to both the genotype of the invading pathogen and the component of infection that was measured. We can consider the broader fitness consequences of these patterns in terms of the ecology of infectious disease and the overall impact of fluoxetine on the likelihood of an outbreak occurring [28], as well as the evolutionary trajectory that a pathogen may take as a result [32,52,70]. To do so, we first parameterized an epidemiological model of infection dynamics to derive two estimates of pathogen fitness in each fluoxetine treatment: the rate at which new infections would be created (*σ*, the composite transmission rate) and the potential for a pathogen to spread through an entirely susceptible population under each condition (*R*_0_, the basic reproductive number). Both metrics of pathogen fitness and invasion capture different aspects of possible infectious disease dynamics, particularly the role of pathogen virulence and the duration of infection (*σ* only) versus that of host demography and population growth (*R*_0_ only).

**Figure 3.**
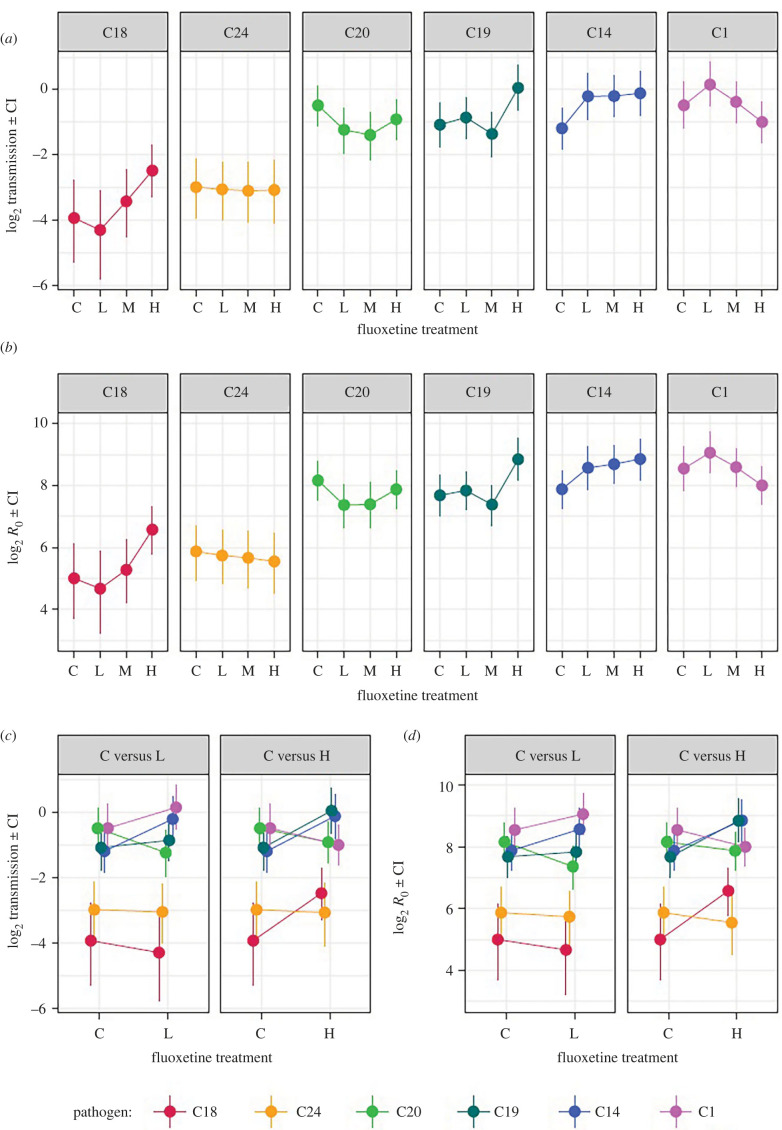
The effect on fluoxetine exposure on (*a*) transmission rate *σ* and (*b*) the basic reproductive number (*R*_0_) for six pathogen genotypes (C18, C24, C20, C10, C14 and C1). Freshwater control (0 ng l^−1^), low (measured concentration: 12 ng l^−1^), medium (114 ng l^−1^) and high fluoxetine (959 ng l^−1^) treatments are denoted as C, L, M and H, respectively. Pairwise reaction norms between control and low, and control and high, fluoxetine treatments are shown for (*c*) *σ* and (*d*) *R*_0_. Data displayed include the mean and 90% uncertainty intervals for the derived metrics and are shown on a log_2_ scale to emphasis fold changes in the metrics of pathogen fitness.

Overall, we found that changes in fluoxetine can lead to a twofold change in transmission and *R*_0_ for a pathogen (note log_2_ scale in figure 3), but with no substantial net increase or decrease in these values, owing to the genotype-specific results. Thus, an increase in fluoxetine does not necessarily act to accelerate the overall spread of disease, but rather results in rank order changes in pathogen fitness and the likelihood of an outbreak occurring. This effect is maximized at higher concentrations of fluoxetine. A comparison of transmission and *R*_0_ between control and low fluoxetine concentration (figure 3*c*,*d*), for example, showed very little difference in fitness values between control and low treatments (nearly all posterior differences ± 90% CI overlap zero, electronic supplementary material, table S2). By contrast, the reaction norms comparing control and high fluoxetine concentrations display far more rank order changes, with three pathogen genotypes all significantly increasing their transmission and *R*_0_ likelihoods at this high concentration (C14, C18 and C19: posterior differences ± 90% CI greater than zero, electronic supplementary material, table S2), and the others trending towards a decline (electronic supplementary material, table S2).

The striking similarity between transmission and *R*_0_ arose because the concentration of fluoxetine was found to have no effect on the overall fecundity, lifespan and intrinsic growth of unexposed hosts, and did not influence the supply of susceptible hosts (which only *R*_0_ captures). The impact of fluoxetine thus appears to be mediated solely through dynamics of infected individuals. This contrasts with more conventional pollutants, such as pesticides and heavy metals, which are frequently reported to increase mortality of various animal species [26,27] as well as disrupt reproduction [25], therefore shaping the overall density or growth rates of susceptible hosts in a population. In the same study system, for example, exposure to the pesticide carbaryl is known to increase mortality and decrease reproductive output [47]. Our results therefore highlight an important distinction between pharmaceutical pollutants and other forms of chemical pollution in their potential influence on host–parasite dynamics. Unlike many other environmental contaminants, pharmaceutical compounds are designed to be safe for human consumption, which may result in subtler, sub-lethal effects on wildlife.

### (d) Can the impact of pharmaceutical pollution on infectious disease be predicted?

Our results underscore the complex task of generating predictions for the influence of pharmaceutical pollution on wildlife health and the likelihood of an infectious disease outbreak. Any response to changes in fluoxetine are unlikely to be shared equally among pathogen genotypes, and will not manifest the same way in every component of host or pathogen fitness. Utilizing only one pathogen, or measuring only a single component of host and pathogen fitness, would have overlooked this important source of variation. For example, if we had only examined the effects of fluoxetine on C1 spore loads, we may have concluded that low levels of fluoxetine increase pathogen fitness, whereas if we had chosen to use only C24, we would have concluded that fluoxetine has no effect at all on disease traits. As a result, simple predictions that exposure to fluoxetine will lead to an unwanted increase in pathogen transmission or a change in the likelihood of an infectious disease outbreak occurring within any population are immediately precluded. Instead, the broader consequences of exposure to fluoxetine for the dynamics of infectious disease centre on more complex changes in the evolutionary dynamics that result from genotype-by-environment interactions.

Genotype-by-environment interactions occur whenever the relative fitness of different host or pathogen genotypes is dependent on the environment in which they are expressed [32,70]. Accordingly, the genotype that is most favoured by selection, as well as the disease characteristics associated with this genotype, can vary depending on the specific environmental conditions a population is currently experiencing. Our results indicate that fluoxetine exposure may result in genotype-by-environment interactions by changing the rank order of pathogen genotypes. This suggests that fluoxetine will influence the maintenance of genetic variation in pathogen populations by modifying which pathogens have the highest fitness, as estimated via transmission or *R*_0_, at any given fluoxetine concentration [34]. The biggest shifts in rank order, however, were observed at the highest concentration of fluoxetine, which is above what is generally detected in the environment [48]. The evolutionary consequences of fluoxetine will thus be sensitive to the concentration of this pollutant in the environment, and the natural variation that is expected following pulse pollution events and seasonal trends [11,71].

## 4. Conclusion

Studies have rarely considered the ecological and evolutionary consequences of pharmaceutical pollutants on disease dynamics (but see [18]). Here we have found that fluoxetine, a widely prescribed psychoactive drug, has little effect on the growth and likely density of susceptible hosts, but instead gives rise to non-monotonic changes in the lifespan and spore proliferation of infected hosts that are highly dependent on a pathogen's genotype. Our parameterized model suggests that fluoxetine as a result is unlikely to affect the overall probability of disease outbreaks in a population, but instead to shift the rank order of pathogen genotypes, enabling the maintenance of genetic diversity [33,34]. This result emphasizes how pharmaceutical pollutants, unlike pesticides or heavy metals [26,27], can have subtle, non-lethal effects on individuals, and require the addition of secondary stressors, such as infection, to fully express their complex effects.

The propensity for fluoxetine to shape pathogen genotype-by-environment interactions (G_P_ × E), particularly at higher concentrations, also hints at a further role fluoxetine may play in shaping the evolution of infectious disease. Both interactions between host and pathogen genotype, and their modification by the environment, are an essential part of host–pathogen evolutionary theory and commonly observed for many components of host and pathogen fitness [45,72]. By focusing here on only pathogen genetic variability, our study has captured only part of the complex way in which pharmaceuticals can potentially interact with hosts and pathogens. Various exposure concentrations, genotypes of both hosts and pathogens, as well as potential effects on free-living stages of pathogens (see discussion in [73]), will need to be considered to fully understand the scope for pharmaceuticals to shape the coevolution of hosts and pathogens in natural populations.

## Data Availability

Data are available from the Dryad Digital Repository: https://doi.org/10.5061/dryad.dv41ns21s [74]. Supplementary material is available online [75].
